# Efficacy of MSC in Patients with Severe COVID-19: Analysis of the Literature and a Case Study

**DOI:** 10.1093/stcltm/szac067

**Published:** 2022-10-01

**Authors:** Martin Grumet, Jason Sherman, Barry S Dorf

**Affiliations:** W. M. Keck Center for Collaborative Neuroscience, Rutgers Stem Cell Research Center, Department of Cell Biology & Neuroscience, Rutgers University, Piscataway, NJ, USA; W. M. Keck Center for Collaborative Neuroscience, Rutgers Stem Cell Research Center, Department of Cell Biology & Neuroscience, Rutgers University, Piscataway, NJ, USA; W. M. Keck Center for Collaborative Neuroscience, Rutgers Stem Cell Research Center, Department of Cell Biology & Neuroscience, Rutgers University, Piscataway, NJ, USA; Department of Medicine, North Shore University Hospital, Manhasset, NY, USA

**Keywords:** COVID, mesenchymal stromal cells, cytokine storm, clinical trials, ARDS, COVID-19

## Abstract

Patients with severe COVID-19 experience cytokine storm, an uncontrolled upregulation of pro-inflammatory cytokines, which if unresolved leads to acute respiratory distress syndrome (ARDS), organ damage, and death. Treatments with mesenchymal stromal cells (MSC) [Viswanathan S, Shi Y, Galipeau J, et al. Mesenchymal stem versus stromal cells: International Society for Cell & Gene Therapy Mesenchymal Stromal Cell committee position statement on nomenclature. *Cytotherapy*. 2019;21:1019-1024] appear to be effective in reducing morbidity and mortality. MSC respond to pro-inflammatory cytokines by releasing anti-inflammatory factors and mobilizing immune cells. We analyzed 82 COVID-19 clinical trials registered at ClinicalTrials.gov to determine MSC dosing, routes of administration, and outcome measures. Nearly all trials described the use of intravenous delivery with most doses ranging between 50 and 125 million MSC/treatment, which overlaps with a minimal effective dose range that we described previously. We also searched the literature to analyze clinical trial reports that used MSC to treat COVID-19. MSC were found to improve survival and oxygenation, increase discharge from intensive care units and hospitals, and reduce levels of pro-inflammatory markers. We report on a 91-year-old man with severe COVID-19 who responded rapidly to MSC treatment with transient reductions in several pro-inflammatory markers and delayed improvement in oxygenation. The results suggest that frequent monitoring of pro-inflammatory markers for severe COVID-19 will provide improved treatment guidelines by determining relationships between cytokine storms and ARDS. We propose that markers for cytokine storm are leading indicators for ARDS and that measurement of cytokines will indicate earlier treatment with MSC than is performed now for ARDS in severe COVID-19.

## Graphical Abstract

**Graphical Abstract:**
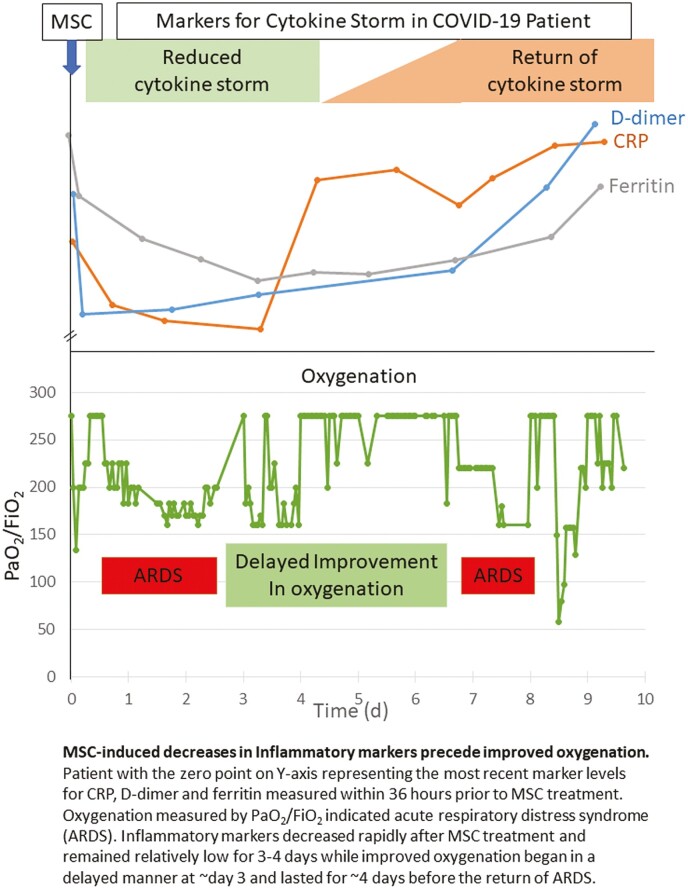

**Lessons Learned**

- The time to start applying MSC therapy should be matched to the severity of the disease.
- MSC disappear rapidly after injection and additional doses may be required for maximal effect.
- The dosage of MSC for therapy needs to be high enough for efficacy but not too high to avoid potential overdoses.

**Significance Statement**

COVID-19 has killed millions of people but there still is no specific treatment for severe COVID-19. Vaccines have lowered the death rate significantly by reducing the percentage of infected patients and the numbers of patients progressing to severe COVID-19. This study shows trends that MSC treatment reduces the rate of death and increases recovery for patients having severe COVID-19. We have analyzed markers of COVID-19 that appear to predict the severity of COVID-19 and more validation is needed. We suggest that these markers provide initial guidelines to optimize when to treat COVID-19 with MSC.

## Introduction

COVID-19 deaths have surged past 6 million worldwide, and there is no sign of an effective treatment for severe disease. A recent review defined mild COVID-19 disease as patients maintaining oxygen saturation (SpO_2_) >90%, moderate disease as patients who required supplemental low flow oxygenation via nasal cannula, and severe disease as patients who required high-flow oxygenation or intubation with mechanical ventilation.^1^ The most effective method so far to prevent death is to prevent infection with vaccines that have proven to be effective, but many people worldwide will probably not get vaccinated until the end of 2022 or beyond. Most treatments tested so far are effective at slowing disease progression at early stages but have limited effects once patients progress to severe COVID-19.^2^ These treatments include monoclonal antibodies (eg, Molnupiravir from Regeneron), convalescent serum,^3^ remdesivir,^4^ anti-IL-6 tocilizumab,^5^ dexamethasone^6^, and paxlovid (Pfizer). However, more effective therapies are needed to treat severe COVID-19.

Cytokine storm is an uncontrolled imbalance of cytokines in which pro-inflammatory overpower anti-inflammatory cytokines and promote pro-inflammatory cellular responses.^7^ If cytokine storm in acute respiratory distress syndrome (ARDS) is not resolved, it often leads to organ damage and death. ARDS is a major risk factor for COVID-19 patients. Cytokine storms that occur in graft versus host disease (GvHD) and sepsis are also associated with a high incidence of morbidity and mortality. Dexamethasone and anti-IL-6 are partially effective in GvHD^8^ and CAR-T-associated cytokine storms.^9^ However, there is no approved treatment for cytokine storms in ARDS.

Mesenchymal stromal cells (MSC) are being investigated for various clinical indications because of their broad anti-inflammatory actions to suppress cytokine storms.^10,11^ MSC are approved to treat steroid-resistant GvHD in Japan and the cells reduced mortality in patients in pediatric care.^12^ In a phase I/IIa clinical trial (NCT04355728) significant survival benefit in moderate to severe COVID-19 patients was observed after 2 doses of 100 million placenta-derived MSC separated by 3 days.^13^ This MSC treatment reduced levels of several pro-inflammatory cytokines including IL-6, IFNγ, and TNFα, thereby mitigating cytokine storm. Athersys, Inc. has reported that multistem mitigated the severity of ARDS but they did not reach their primary outcome,^14^ and at least one other company-sponsored clinical trial is in progress (NCT04456439).

Studies of mechanisms of activated MSC in vitro and in animal models of inflammatory diseases have implicated secretion of anti-inflammatory factors including PDGE2 and IL-10.^15,16^ Thus, pro-inflammatory cytokines that are elevated in cytokine storm circulate systemically in the blood and activate MSC delivered intravenously to secrete anti-inflammatory factors that can mitigate cytokine storm and reduce key inflammatory markers such as C-reactive protein (CRP).^17,18^

Here we analyze publications on COVID-19 clinical trials and case reports of MSC therapy in patients with severe COVID-19 who were hospitalized. The oldest patient with severe COVID-19, so far, that has been reported to survive after MSC treatment with decreased levels of proinflammatory cytokines was 79 years old.^17^ We report that a 91-year-old male with severe COVID-19 responded transiently to a single dose of 300 million MSC with rapidly reduced levels of several proinflammatory markers of cytokine storm and delayed improvement in oxygenation.

## Materials and Methods

We performed 2 separate analyses for MSC treatments of COVID-19 patients. First, we created a database of current clinical trials listed on ClinicalTrials.gov using the search terms mesenchymal and COVID-19. Second, we searched PubMed using the same terms MSCs and COVID-19 to identify publications that reported the use of MSCs in COVID-19 patients. Searches were performed in October 2021.

In study 1, we collected data from ClinicalTrials.gov, using a combination of 2 keywords—mesenchymal and COVID-19, which yielded 97 clinical trials. A total of 15 trials were excluded as they were not MSC-specific treatments, including 7 that used exosomes, 6 that did not use MSC treatments, and 2 that were considered as medical tourism (Supplementary Table S1). Data obtained directly from ClinicalTrials.gov included NCT number (a unique identifier for each trial), intervention, age, sponsor, gender, enrollment, and start/end dates that were exported as an Excel document. Each clinical trial record was analyzed manually to collect more critical data including dose, repeated dose, route of delivery, and effects of treatment, as performed previously.^19^ Two studies reported on 28^20^ and 66 trials^21^ registered in ClinicalTrials.gov using MSC in COVID, respectively; however, the search terms were not described in either case. These papers include informative discussions of potential mechanisms of MSC action in COVID-19^21^ and considerations of various aspects of MSC action in these trials.^20^

Study 2 focused on clinical trials and case studies by searching PubMed with the terms mesenchymal and COVID-19. Papers that reported results on completed trials containing survival data as well biomarkers of disease were included. Although reporting on parameters of COVID-19 disease vary widely, most reported decreases in CRP, which is a widely used clinically relevant marker associated with inflammation in a wide range of inflammatory disorders. Normal levels for CRP range from 3 to 10 mg/L^22^) and any patient whose CRP levels were <10 mg/L were excluded as they were not considered in a cytokine storm. Fewer studies reported on other clinical markers associated with inflammation including D-dimer, ferritin, and IL-6.

ARDS is measured using the Berlin 2012 ARDS diagnostic criteria.^23^ It is rated as mild, moderate, and severe by the levels of the PaO_2_/FiO_2_ oxygenation index. PaO_2_ is the arterial partial pressure of O_2_, FiO_2_ is the fraction (%) of inspired O_2_ that the patient is receiving, and the ratio is a dimensionless factor that is used to describe ARDS. A mild score ranges from 200 to 300, moderate is 100 to 200, and severe ARDS is below 100. A healthy individual will have a score above 300 (usually 400-500) or be characterized by a SpO_2_ level of at least 98%. When infected with COVID-19, patients will likely require oxygen supplementation, which is expected to be reduced by treatment with MSCs when effective. Some papers only reported scores as % oxygen.

### Assays for Markers of Inflammation and Disease

ELISA assays for CRP, D-dimer, and ferritin were performed as described at Northwell Health, Manhasset, NY.^24^ Cytokine storm was defined using a combination of disease and inflammatory markers with thresholds as follows: ferritin, >0.3 mg/L; CRP, >30 mg/L; and D-dimer >0.5 mg/L.

## Results

### Study 1: MSC Treatment in COVID-19 Clinical Trials

**Figure 1.**
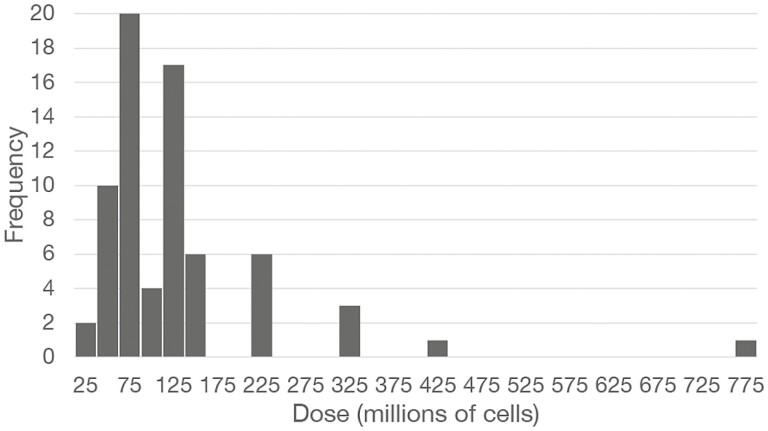
Frequencies of MSC doses for clinical trials. Numbers of MSC in clinical trials with COVID-19 patients are plotted for each dose. Each bin is 25 million MSC.

To analyze clinical trials planned, data were collected from ClinicalTrials.gov, which is the largest and most often used database for recording clinical trials worldwide. A combination of 2 keywords—mesenchymal and COVID-19, yielded 82 MSC trials, of which 73 trials reported dosing (Supplementary Table S1). In an analysis of hundreds of clinical trials using MSC, minimal effective intravenous injection (IV) doses for MSC treatment were determined to range from 70 to 140 million cells per dose.^19^ In the current study, 14 trials indicated doses below 70 million cells ranging from 2 to 50 million in a single treatment, which are near or below the threshold for a minimal effective dose.^19^ Eleven studies used doses ranging from 200 to 750 million cells per single treatment, with the highest possibly an overdose because effects of MSC may follow an inverse U-shaped curve.^25^ The most frequently used range was 50-125 million cells per dose (Fig. 1), which overlaps with the previously determined minimal effective dose range for MSC delivered by IV of 70-140 million cells per dose.^19^

Most trials in Supplementary Table S1 are phase I, I/II, or II, and one is a phase III. Few reported results except for NCT04339660,^18^ NCT04355728,^13^ NCT04348461,^26^ and NCT04367077,^14^ which are discussed below.

Although IV is the least invasive method, MSCs are mostly cleared from the circulation after IV injection and accumulate initially in the lungs, and subsequently in the liver, spleen, kidney, and bone marrow within 48 h.^27^ The rapid clearance of the MSC may be one reason why multiple doses are given in many trials at intervals of 1-7 days after the first injection (Supplementary Table S1). There are several other reports of MSC in severe COVID-19 trials with limited data but in the next section only case studies are analyzed in which data were provided on individual patients before and after MSC treatment.

### Study 2: COVID-19 Patients Treated with MSC

We analyzed multi-patient trials that reported at least 70% survival (Table 1). These trials used a minimal of ~70 and a maximum of 300 million cells/patient. In a double-blind, randomized, and controlled COVID-19 clinical trial, patients treated intravenously (IV) with 2 doses of umbilical cord derived-MSC on days 0 and 3 showed significant reductions in the concentrations of pro-inflammatory IFNγ, IL-5, IL-6, IL-7, TNFα, and TNFß at a single time-point 6 days after treatment by comparison to the day prior to treatment.^13^ This suppression of cytokine storm was accompanied by significantly improved survival (91% with MSC treatment vs. 42% in the control group). However, this study did not report on CRP, the widely used clinical COVID-19 marker. In contrast to this high level of survival (88%) the survival rate for severe COVID-19 is typically ~50%^28^ among comparable patients treated with similar standards of care during the early stages of the COVID-19 pandemic. Three other trials reported improvement to ~70% survival but no controls were included to evaluate significance. An uncontrolled MSC compassionate-use study using IM delivery of PLX cells reported survival at 28 days in 7/8 severe COVID-19 patients,^17^ which is similar to the survival rate reported by Lanzoni et al., 2021. Daily data on CRP and oxygenation was reported in Barkama et. al. but not in the other 4 studies making the early kinetics of MSC effects unclear (Table 1). Trials using MSC in severe COVID-19 patients where individual patient data were not reported over the first few days of treatment were excluded.^26,29^

**Table 1.** Multi-patient COVID-19 clinical trials. The effects of MSCs in 5 clinical trial papers are summarized. After infusion of MSCs, either intravenously (IV) or intramuscularly (IM) the effects were analyzed using 3 criteria: CRP levels, oxygenation efficiency, and clinical outcomes. Individual patient data were not available. ND, not done.

Authors	Cell	Severity	Route	Dose (millions of cells)	C-reactive protein	Oxygenation	Clinical outcomes
Bakarma et al. (2020)	Placenta PLX-PAD	Critically ill on mechanical ventilation	IM	300	Decreased 38% at d3 77% at d5 (*P* < .002)	Significant improvement at 5 days (P < .05)	5 Discharged 7/8 Recovered
Sadeghi et al. (2021)	Placenta	Severe with COVID-associated pneumonia	IV	1-2/kg	Decreased in 6 timing variable	6 patients improved, timing variable	7/10 Recovered 3 Died
Sanchez et al. (2020)	Adipose MSC	Critically ill on mechanical ventilation	IV	1/kg	Decreased in 85% of patients	ND	9/13 Improved
Leng et al. (2020)	Umbilical	Mixed	IV	1/kg	Unspecified decrease	Significant improvement in 2 days	7/10 Improved 3/7 Discharged
Lanzoni et al. (2020)	Umbilical With a control group	Mixed	IV	100	ND	ND	Survival 91% +MSC vs. 42% -MSC (*P* < .015)

**Figure 2.**
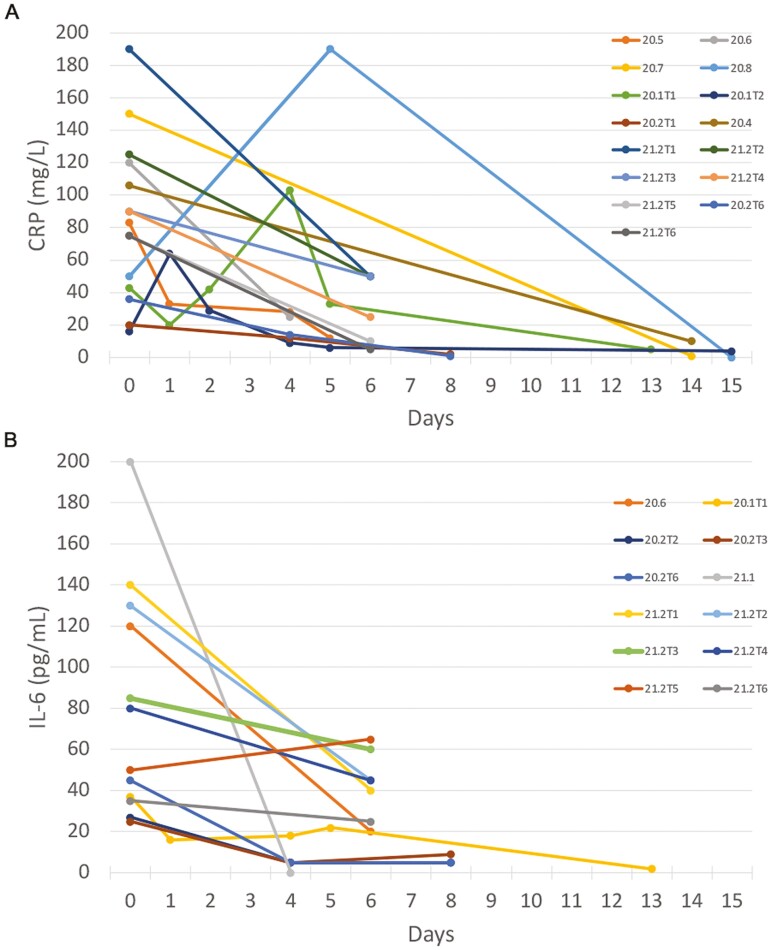
Expression levels of CRP and IL-6 in individual COVID-19 patients over time. The zero-time point represents the closest pre-MSC treatment level of expression for CRP (**A**) and IL-6 (**B**). There are fewer numbers of trials for IL-10 than for CRP because they did not all report data for IL-6. All points are single measurements.

A major goal of this study was to identify early markers that correlate with COVID-19 responsiveness to MSC over time, but such data were only reported by Barkama et al. in their multi-patient trial only for CRP and oxygenation. Therefore, we searched for publications of case studies that included data on several parameters including oxygenation, survival, and expression of CRP, D-dimer, and IL-6. Few trials reported on all these disease markers. The largest number of trials (15) reported data on CRP. There was a trend of decreasing expression after a first MSC treatment (Fig. 2A). A total of 12 trials reported data on IL-6 and, all but 1 showed a decrease after MSC treatment (Fig. 2B). Of these, 11 reported improved oxygenation (Table 2).

**Table 2.** Data on patient disease severity and oxygenation. For ARDS, oxygenation reported as PaO_2_/FiO_2_ was considered to be normal above 300, mild for 200-300, moderate for 100-200, and severe for <100. In some cases, 98% SpO_2_ on room air was reported as a normal level of ARDS. The MSC doses for 20.7, 20.8, and 20.1 were 10^6/^kg,^30-33^ and for the 4 patients designated 20.4 was 30 × 10^6^ with 2 additional doses at 3-day intervals.^33^ Patients designated 21.2 received 200 × 10^6^ MSC with 2 additional doses at 3-day intervals. Note a dose of 10^6/^kg for a 70 kg patient is 70 × 10^6^ MSC.

Trial	ARDS before	ARDS after
20.7	Severe	Normal
20.8	Moderate	Normal
20.1T1	Severe	Moderate
20.1T2	Moderate	Normal
20.4	Severe	Normal
21.2T1	Severe	Normal
21.2T2	Moderate	Normal
21.2T3	Severe	Normal
21.2T4	Severe	Normal
21.2T5	Moderate	Normal
21.2T6	Severe	Normal
20.9	Severe	Severe with transient improvement

These results suggest that decreases in CRP and IL-6 levels are associated with improved oxygenation. CRP is a marker that is routinely measured in COVID-19 patients in the hospital along with D-dimer and ferritin,^24^ the latter of which has not been reported as widely in COVID-19 cases. This is not to say that other biomarkers may not be better, rather it highlights the potential use of these markers, and the lack of systematic reporting to analyze potential marker correlations with the action of MSC in COVID-19 patients.

### Case Study

**Figure 3.**
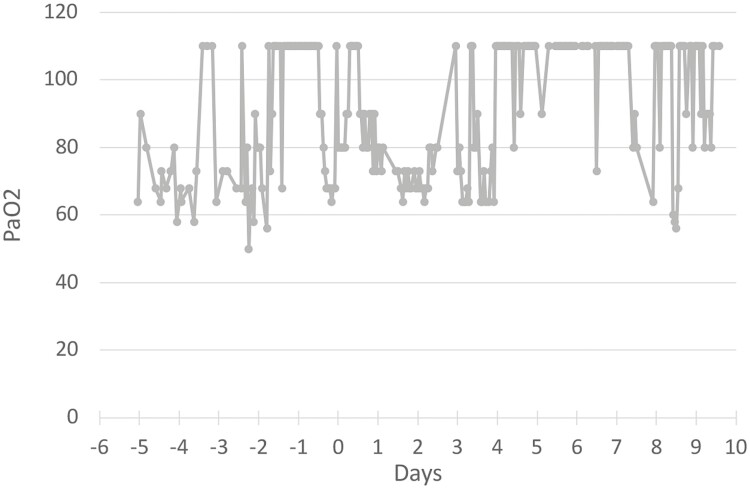
Changes in oxygenation after treatment with MSC. Data for patient 20.9 with the zero-time point representing the time of MSC treatment. Note that the oxygenation measured by PaO_2_ fluctuated primarily between 60 and 90 prior to MSC treatment (negative times), indicative of ARDS. Improved oxygenation (increase in PaO_2_) began in a delayed manner before day 3 after MSC treatment and lasted for approximately 4 days before the deceasing sporadically.

A 91-year-old male (patient 20.9) was admitted to Long Island Jewish Hospital on October 16, 2020 with a SpO_2_ of 80% and double pneumonia indicating a severe case of COVID-19. His percent oxygenation (PaO_2_) fluctuated between 60% and 80% (Fig. 3). He had highly elevated pro-inflammatory markers, including CRP, D-dimer, and ferritin, indicating a cytokine storm. Prior to admission, the patient was treated with a single bolus of 400 mg hydroxychloroquine. In the hospital, patient 20.9 was treated with the standard of care for LIJ Hospital, at that time consisting of remdesivir, dexamethasone^34^, and convalescent serum. After 3 days on an oxygen cannula, the patient’s PaO_2_ continued to be dangerously low, and he was moved to the intensive care unit (ICU) and intubated 18 h later. After 32 h, patient 20.9 received a total dose of 300 million PLX PAD cells distributed in 15 IM injection sites as described.^17^

**Figure 4.**
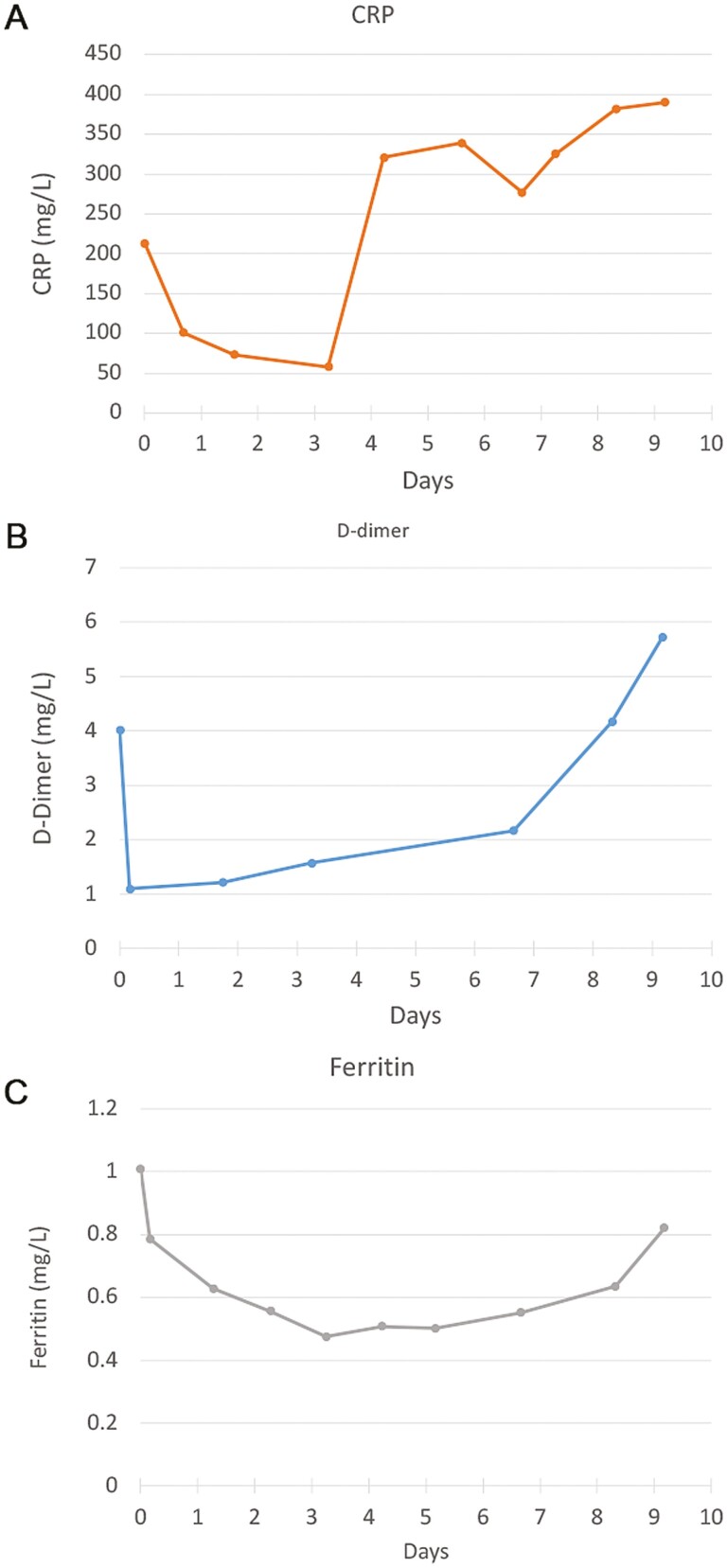
Changes in markers for cytokine storm after treatment with MSC. Data for patient 20.9 with the zero-time point on the *Y*-axis representing the most recent marker levels for CRP, D-dimer, and ferritin measured within 36 hours prior to MSC treatment. These inflammatory markers decreased rapidly after MSC treatment and remained relatively low for 3-4 days after which they rose at different rates. All points are single measurements.

Within the next few days, there were marked decreases in 3 well-recognized inflammatory markers (Fig. 4). The data for all these parameters were single measurements at the indicated time points, thus no statistical analyses could be performed to analyze the data. Ferritin levels dropped by 22% after 4 h and by 51% after 4 days, and D-dimer levels dropped by 73% soon after treatment, suggesting a rapid response to MSC. CRP levels dropped by 53% within 16 h and by 73% after 4 days. The decreased levels of all these pro-inflammatory markers suggest that anti-inflammatory effects occurred in this patient within 16 h after MSC administration and persisted for ~4 days at least for CRP.

During the 2 days after MSC treatment, the patient’s PaO_2_/FiO_2_ remained low at ~170 and then improved to fluctuate near ~270 for the next 6 days (Supplementary Fig. S1). Decreases in CRP, ferritin, and D-dimer were observed within 1 day after MSC treatment, while improvement in PaO_2_/FiO_2_ appeared to lag behind until the third day (Fig. 4 and Supplementary Fig. S1). However, there was a dramatic jump in CRP on the fourth day after injection, suggesting that the effect of PLX treatment was subsiding. This is consistent with reduced efficacy of PLX after ~4 days (Racheli Ofir, Pluristem, personal communication). D-dimer levels began to rise again to dangerous levels on the 8th day. These results suggest that MSC treatment mitigated cytokine storm rapidly and then improved the physiological state of the patient with approximately a 3-day delay. The patient received a second total dose of 300 million PLX PAD cells on day 7 but there did not appear to be any response (Supplementary Fig. S1). The patient developed a bacterial infection on day 9 after treatment and died the next day.

## Discussion

There are 54 clinical trials with MSC for severe COVID-19, which represent the largest subgroup of all MSC trials registered during the past 2 years, indicating substantial interest in this new therapy. Clinical outcome measures including survival and discharge from ICUs and hospitals are usually reported as measures of MSC efficacy in severe COVID-19. Doses indicated for trials cover a wide range from 5 to 750 million cells/dose with the large majority falling in the range of 50-125 million MSC/dose (Fig. 1), which overlaps with a minimal effective IV dose range of 70–140 million proposed previously.^19^ There is concern that 31 COVID-19 trials may be administering relatively low doses in the range between 50 and 75 million MSC, which may be borderline to obtaining robust effects. In approximately half of all patients treated so far, at least 1 additional dose has been administered and this may increase the effect by prolonging treatment.

Inclusion criteria for clinical trials are often broad including a range of COVID19 disease severities from mild to severe based on pulmonary parameters. More research is necessary to establish well-defined markers as molecular signatures for severe COVID-19. These markers would be most useful if they predict the efficacy of treatment over time as well as indicate times at which additional treatments should be administered. We believe that the transition of a patient into a cytokine storm is likely to be an early indication of impending ARDS and severe COVID. Given that MSC treatment plays a critical role in suppressing cytokine storm in various inflammatory disorders including GvHD and ARDS,^11^ we need to define key markers of cytokine storm that can be used to track the severity of COVID-19 disease and then follow reductions in these markers to assess the response to various treatments.

The case study presented here shows that even in a 91-year-old patient with critical COVID-19 disease MSC reduced disease markers including CRP, ferritin, and D-dimer transiently. However, on the 4th day after IM MSC injection, CRP jumped to >300 mg/L and remained high despite a second MSC dose given on day 7. In contrast, in 7/8 patients that received similar treatment, CRP levels remained low, and patients improved.^17^ We will use the 20.9 case as a springboard to discuss the usefulness of markers expressed over time because in this case marker data were collected almost daily unlike nearly all previous reports in which only one or a few data points were reported after MSC treatment. The following markers have been found to be indicators of cytokine storm in COVID-19 but there is minimal data on their expression over time.

### C-Reactive Protein (CRP)

CRP, which is an acute phase serum protein induced by various inflammatory mediators such as IL-6, activates macrophages via a CRP receptor. CRP is widely used as a marker for inflammation in GvHD, sepsis, and COVID-19. In a large study of 5700 COVID-19 patients, the CRP threshold for cytokine storm was determined to be 30 mg/L.^24^ Increased CRP levels are one of the most highly correlated markers with severe COVID-19^35^ and were found to decrease in 13/15 COVID-19 cases treated with MSC (Fig. 2A), and in several multi-patient trials.^13,17,30,31^ Improved oxygenation was accompanied by decreases in CRP and, in the majority of cases, it was reduced below the 30 mg/L thresholds to near normal levels. CRP decreased transiently in patient 20.9 soon after MSC treatment and jumped 4 days later suggesting reduced efficacy. But it never dropped below 50 mg/L suggesting incomplete suppression of cytokine storm.

### D-dimer

D-dimer, which is a product of fibrin degradation that appears after blood clot destruction due to fibrinolysis, is considered an early marker for inflammation in COVID-19.^35^ D-dimer levels >0.5 mg/L are considered abnormally high.^36^ D-dimer levels decreased from a high of ~4 mg/L in patient 20.9 soon after MSC treatment but increased 8 days later. But it never dropped below 0.5 mg/L suggesting incomplete suppression of cytokine storm.

### Ferritin

Ferritin, which was initially described as an acute phase protein accompanying viral infections, is an early indicator of inflammation.^28^ Several cytokines such as IL-6 were found to stimulate the release of ferritin into the blood. Many laboratories consider serum ferritin levels greater than 0.2 mg/mL in women and greater than 0.3 mg/mL in men to be abnormal.^37^ Ferritin levels decreased in patient 20.9 soon after MSC treatment and remained relatively low until day 9 when it increased dramatically but never dropped below 0.4 mg/mL, suggesting incomplete suppression of cytokine storm.

### IL-6

IL-6 is a pro-inflammatory cytokine that is expressed early along with other cytokines in response to viral infections and it is associated with cytokine storm in severe COVID-19.^38,39^ IL-6 induces expression of CRP, and antibodies against IL-6 reduced disease severity making it a functional biomarker. Dexamethasone alone or in combination with IL-6 inhibitors tocilizumab or anakinra reduced levels of markers associated with cytokine storm including CRP, and improved survival.^24^ Thus, IL-6 is an early marker for severe COVID-19 that is also an important target for therapy. However, IL-6 was not measured in blood taken from patient 20.9.

Most COVID-19 patients that develop severe disease associated with cytokine storm require ventilation assistance and have a high risk of organ damage and death. In severe COVID-19, it is likely that cytokine storm, ARDS, and coagulation disorders are the major risk factors as compared to the viral load, which decreases in <1 week after the onset of symptoms. Thus, it is probably critical to treat as early as possible with MSC or other therapies that target cytokine storm when it is first detected.

### Cytokine Storm and COVID-19 Patient Survival

In addition to the trials described in Table 1, other reports included combinations of severe and non-severe COVID-19 patients or presented limited data or minimal improvement. In one, survival was only 50% with MSC, which is what most studies find as the level without MSC treatment.^40^ In another, only 55% of COVID-19 patients treated by IV injection of MSC every 2 days survived.^41^ An elegant study used genetically-modified Ace2-MSC^42^ and another was also not included because many patients did not receive oxygenation, hence they were unlikely to be severe COVID-19.^29^ A recent randomized, single-blind, placebo-controlled (29 patients/group) phase II clinical trial reported that both severe and critical patients treated with MSCs showed improvement by day 7 with shorter hospital stays, less time required for the remission of symptoms, and reduced levels of CRP and pro-inflammatory cytokines including IL-6, but it did not report on survival.^18^ A clinical trial with 4 patients showed improved oxygenation and decreases in both CRP and IL-6 after MSC treatment, but no timing of the response was reported.^43^ In summary, there is a trend toward improvement in severe COVID-19 with single doses of MSC and this trend is clearer with multiple doses. However, few studies reported data daily as presented here for patient 20.9 and in a recent report by Pluristem.^17^

The PLX cells produced by Pluristem are the same cells used to treat patient 20.9. Key inflammatory and disease factors including CRP, D-dimer, and ferritin were reduced in as little as 1 day in patient 20.9, which is earlier than other cases that first reported results at 2-3 days following MSC treatment.^30,31^ In a COVID-19 trial with PLX, average CRP levels of ~220 mg/L were reduced significantly by 45% at 3 days and by 77% to ~50 mg/L at 5 days after IM injection of MSC.^17^ Patient 20.9 showed a similar pre-treatment level and course of decrease to ~50 mg/L on day 3 after treatment, but on day 4 after PLX injection, the CRP level jumped to over 300 mg/L and remained high (Fig. 4A), suggesting that the cells were losing efficacy in suppressing cytokine storm. A second dose given 7 days after the first did not appear to be effective and the patient died 3 days later. Whereas reduction in pro-inflammatory factors occurs transiently for ~4 days after IV MSC injection (eg, on days 0, 2, and 4), IM-delivered MSC may survive and act a few days longer.^25^

Routes of delivery, dose levels, repeat dosing, and interval between repeat doses need to be optimized to yield maximal benefit of MSC therapy in COVID-19 and other diseases in which cytokine storm plays a central role. IV is the most common route of MSC delivery. MSC rapidly accumulate in the lungs and are cleared rapidly to the spleen and liver^27^ with a half-life of ~24 h.^16^ To extend the treatment period, repeat injections of MSC are often performed at intervals of 2-7 days (Supplementary Table S1).^19^ A range of MSC isolated from different sources have been reported to be safe in hundreds of clinical trials^19^ but the state of the MSC may be important for their efficacy.^25^ Considering that cytokine storm is a major target for MSC in clinical trials in GvHD and non-COVID-19 ARDS, the collective results stemming from optimization of MSC therapy are likely to help design improved COVID-19 trials.

Another important consideration in choosing MSC for therapy delivered by IV is the level of expression of the coagulation factor TF/CD142. Bone marrow MSC have relatively low expression while some other sources of MSC have higher expression increasing the risk of clotting in general for MSC, and in particular, for COVID-19.^44,45^ This appears to be less of a problem when MSC are administered in tissue such as IM, which is the route used by Pluristem and for patient 20.9.

The responsiveness to MSC reported here for a 91-old patient with severe COVID-19 may justify the expansion of treatment options to patients beyond the typical upper age limit in clinical trials of ~65-80 depending on the patient’s health status. Patient 20.9 may have benefited further from the second treatment had it occurred after ~4 days rather than after 7 days. In addition, patient 20.9 may have benefited further if he had received the first MSC treatment before intubation at a time when he was in a cytokine storm already for several days. In the future, it should be determined at what time to treat so that MSC will blunt cytokine storm as soon as possible to prevent progression to organ failure and death.

Differential co-expression networks in monocytes of COVID-19 patients showed dramatic changes 2 days after MSC injection that returned to the pretreatment pattern within 4 days.^46^ This is additional evidence indicating that MSC have transient effects to modulate monocytes even though physiological improvement can continue for days to weeks following treatment. Transient effects on immune cells justify serial IV dosing at intervals of ~3-4 days. Longer intervals between IM doses may be effective given longer survival times of MSC in tissues versus after IV injection.^25^

## Conclusion

Several well-documented markers are available to detect the early stages of cytokine storm and they should be used to develop criteria for when to begin MSC treatment in COVID-19 disease. We suggest that at the first clear sign of cytokine storm, ie, the sufficient elevation of at least 2 key inflammatory markers including IL-6 and CRP, or ferritin or D-dimer in patients requiring supplemental oxygenation, would be the time to treat with MSC or some other inhibitor of the cytokine storm. Dexamethasone is given widely to prevent the development of cytokine storms, but it is often not effective by itself.^24^ Data reviewed here indicate that robust responses could be observed as early as 1 day after MSC injection, thus it is important to measure key markers of cytokine storm early and daily, if possible. Monitoring the same markers should continue for several days in new clinical trials to determine when MSC suppresses cytokine storm. The time at which markers for cytokine storm begin to rise again is likely to be an optimal time to deliver additional doses. Based on limited data summarized here it is likely that a second dose will be needed ~4 days after IV delivery and perhaps longer delay times after IM injections of MSC, which have a longer half-life in vivo. Alternatively, a longer-lasting therapy using encapsulated MSC could provide much longer-term treatment by protecting the survival of MSC in vivo for several months.^47-49^ It will be most valuable to follow these markers over time in individual patients both in clinical trials and then in practice. This is a rational approach to using MSC therapy for COVID-19 and it will probably be useful for other acute diseases involving cytokine storm including sepsis, GvHD, and non-COVID-19 ARDS.

Finally, it is remarkable that patient 20.9, who is the oldest patient with severe COVID-19 receiving MSC therapy described to date, responded to treatment with the lowering of several key proinflammatory markers and improved oxygenation, indicating partial suppression of cytokine storm. Unfortunately, case studies typically report success but not failures; thus, it is critical to perform placebo-controlled studies and report negative as well as positive results to prevent publication bias that may favor positive outcomes.

## Supplementary Material

szac067_suppl_Supplementary_Table

szac067_suppl_Supplementary_Figure

## Data Availability

The data that support the findings of this study are available from the corresponding author upon reasonable request.
