# Supplementary figures and images for: Efficacy of MSC in Patients with Severe COVID-19: Analysis of the Literature and a Case Study
Source: Stem Cells Transl Med. 2022 Oct 1;11(11):1103–12. doi: 10.1093/stcltm/szac067 (PMC9672850; doi:10.1093/stcltm/szac067)

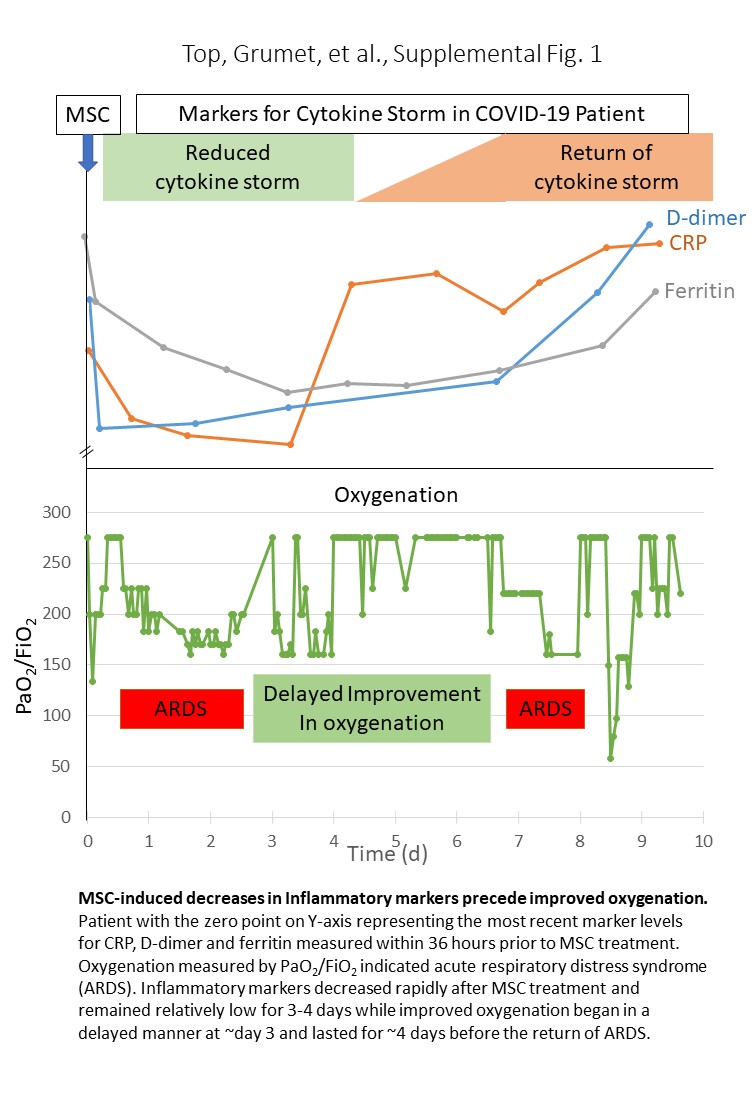

Supplement: szac067_suppl_Supplementary_Figure [file szac067_suppl_supplementary_figure.jpeg]
